# Pre-Clinical Evaluation of a Replication-Competent Recombinant Adenovirus Serotype 4 Vaccine Expressing Influenza H5 Hemagglutinin

**DOI:** 10.1371/journal.pone.0031177

**Published:** 2012-02-17

**Authors:** Jeff Alexander, Simone Ward, Jason Mendy, Darly J. Manayani, Peggy Farness, Jenny B. Avanzini, Ben Guenther, Fermin Garduno, Lily Jow, Victoria Snarsky, Glenn Ishioka, Xin Dong, Lo Vang, Mark J. Newman, Tim Mayall

**Affiliations:** PaxVax Inc., San Diego, California, United States of America; University of Georgia, United States of America

## Abstract

**Background:**

Influenza virus remains a significant health and social concern in part because of newly emerging strains, such as avian H5N1 virus. We have developed a prototype H5N1 vaccine using a recombinant, replication-competent Adenovirus serotype 4 (Ad4) vector, derived from the U.S. military Ad4 vaccine strain, to express the hemagglutinin (HA) gene from A/Vietnam/1194/2004 influenza virus (Ad4-H5-Vtn). Our hypothesis is that a mucosally-delivered replicating Ad4-H5-Vtn recombinant vector will be safe and induce protective immunity against H5N1 influenza virus infection and disease pathogenesis.

**Methodology/Principal Findings:**

The Ad4-H5-Vtn vaccine was designed with a partial deletion of the E3 region of Ad4 to accommodate the influenza HA gene. Replication and growth kinetics of the vaccine virus in multiple human cell lines indicated that the vaccine virus is attenuated relative to the wild type virus. Expression of the HA transgene in infected cells was documented by flow cytometry, western blot analysis and induction of HA-specific antibody and cellular immune responses in mice. Of particular note, mice immunized intranasally with the Ad4-H5-Vtn vaccine were protected against lethal H5N1 reassortant viral challenge even in the presence of pre-existing immunity to the Ad4 wild type virus.

**Conclusions/Significance:**

Several non-clinical attributes of this vaccine including safety, induction of HA-specific humoral and cellular immunity, and efficacy were demonstrated using an animal model to support Phase 1 clinical trial evaluation of this new vaccine.

## Introduction

Since 1996, it has been reported that several novel avian subtypes, H5N1, H7N1, H7N2, H7N3, H7N7 and H9N2 have crossed the species barrier from domestic poultry to humans and caused a spectrum in severity of human disease, including fatalities [1], [2], [3], [4]. H5N1 influenza virus is of special concern due to several factors including its endemic hold in poultry populations in Southeast Asia, a spread to at least 60 countries, and a case fatality rate of more than 50% upon transmission to humans [5]. At present, transmission among humans is rare but there is the potential for mutational events and/or genetic re-assortment which could result in the evolution of a highly virulent pandemic strain with potentially greater loss of life [6]. The recent outbreak and subsequent pandemic caused by a swine-origin H1N1 influenza virus highlights the real danger regarding emergence of novel influenza strains. Thus, the need for effective influenza vaccines remains a recognized global imperative.

Currently, the only U.S. approved stockpiled H5N1 influenza vaccine is based on virus propagated in embryonated chicken eggs. Several issues limit the effective use of inactivated H5N1 influenza vaccines generated using this strategy: 1) reliance on embryonated chicken eggs; 2) safety concerns of the H5N1 influenza virus grown in bulk before inactivation [7]; 3) delivery of the vaccine by needle; and 4) requirement for two doses to generate significant immune responses in naïve individuals. The current stockpile consists of a vaccine to A/Viet Nam/1194/2004 which is a clade 1 virus. Circulating strains have evolved over time and moved on from clade 1 such that it is entirely possible that the current vaccine would be ineffective against a new emerging strain. Our Ad4 system has the advantage that it can be rapidly switched to another HA type to meet the challenge of an emerging pandemic strain.

We sought to address these concerns and thus based our H5N1 influenza vaccine development on the live, replication-competent, orally administered U.S. military adenovirus serotype 4 (Ad4) vaccine which was administered to more than 10 million U.S. recruits between 1971 and 1999 and shown to be very well tolerated and safe [8], [9], [10]. Importantly, the Ad4 U.S. Military vaccine protects against Ad4 respiratory disease confirming that oral delivery and in vivo replication induce protective immunity against respiratory exposure to adenovirus. This highlights the use of the Ad4 vector platform for vaccine development as having several attributes of an ‘ideal’ vaccine including; safety, delivery without hypodermic syringe and relatively low cost of goods. Also of importance, in the case of influenza pandemics, is the capacity to readily construct adenovirus recombinant vectors encoding genes from newly emerging pandemic viruses to meet vaccine demands of a global threat. Additionally, delivery to and utilization of adenovirus recombinant vector vaccines in remote locations without the aid of cold storage may be feasible. Studies by Alcock and colleagues [11] demonstrated that when adenovirus was slowly dried in the presence of certain sugars, the resulting formulation could then be stored for 6 months, at up to 45°C, with minimal viability loses. Clinical trial evaluation will be required to evaluate other ideal parameters such as induction of protective immune responses following only one immunization.

Adenoviruses (Ad) are non-enveloped DNA viruses that have been extensively studied as recombinant vector vaccines for various viral, bacterial and parasitic disease agents or indications including; HIV, Dengue, Rabies, Ebola, Japanese Encephalitis virus, HBV, *Streptococcus pneumonia*, Tuberculosis, and Malaria [12], [13], [14], [15], [16], [17], [18], [19], [20], [21], [22]. Several attributes of the Ad vector make it potentially useful for vaccine development: 1) growth to high titer in cell culture, especially A549 cells; 2) ability to infect dividing and non-dividing host cells; and 3) stimulation of both the innate and adaptive immune systems via induction of pro-inflammatory cytokines and chemokines [23], [24], [25], [26], [27], [28]. Studies using non-replicating Ad-vectored influenza vaccines have demonstrated the utility of this approach with documented levels of immunogenicity and efficacy using animal models [29], [30], [31], [32], [33], [34], [35]. It should be noted that there are potential limitations for use of adenovirus vectors in vaccine development. A common concern is the existence of pre-existing antibodies to adenovirus, likely due to exposure during childhood and development of neutralizing antibodies against several adenoviruses, which could inhibit transgene-specific immune responses [36]. In regard to use of replicating adenoviruses as vaccine vectors, there are restrictions such as a limited cloning capacity of 3 to 4 kb for transgene inserts [37].

As the next step in the development of influenza vaccines, we anticipated that the use of mucosally-delivered, replication-competent Ad4 vectored vaccines would have advantages relative to non-replicating Ad vectored vaccines including: 1) use of lower doses; 2) simulation of natural infection and thus induction of potent immune system responses (innate, humoral and cellular) and 3) induction of immunological memory [28], [37], [38], [39], [40].

In this pre-clinical study, an experimental replicating Ad4-H5-Vtn influenza virus vaccine was designed, produced, and evaluated for safety, immunogenicity and efficacy using an established murine model with the goal of generating data to support Phase 1 clinical trial evaluation of this vaccine.

## Materials and Methods

### Ethics Statement

All animal procedures were performed at Absorption Systems, San Diego CA. The Institutional Animal Care and Use Committee at Absorption Systems approved this study (Protocol #VC-11-08-139). Murine studies were completed in accordance with the NIH “Guide for the Care and Use of Laboratory Animals.” Absorption Systems has the following accreditations: Association for Assessment and Accreditation of Laboratory Animal Care, NIH Assurance # A4282-01, USDA Registration # 93-R-0444.

### Cell lines

The type and source of the cell lines used are as follows: A549 (human epithelial lung carcinoma cell line; ATCC #CCL-185, Manassas, VA); MRC-5 (human embryonic lung fibroblast cell line; ATCC #CCL-171); HepG2 (human hepatocellular carcinoma cell line; ATCC #HB-8065); Hu Tu 80 (human duodenum adenocarcinoma cell line; ATCC #HTB-40); H1299 (human lung carcinoma cell line; ATCC #CRL-5803); and MDCK cells (canine kidney cell line; ATCC #CCL-34).

### Construction, Production and Purification of Ad4-H5-Vtn Vector Virus

**Figure 1:**
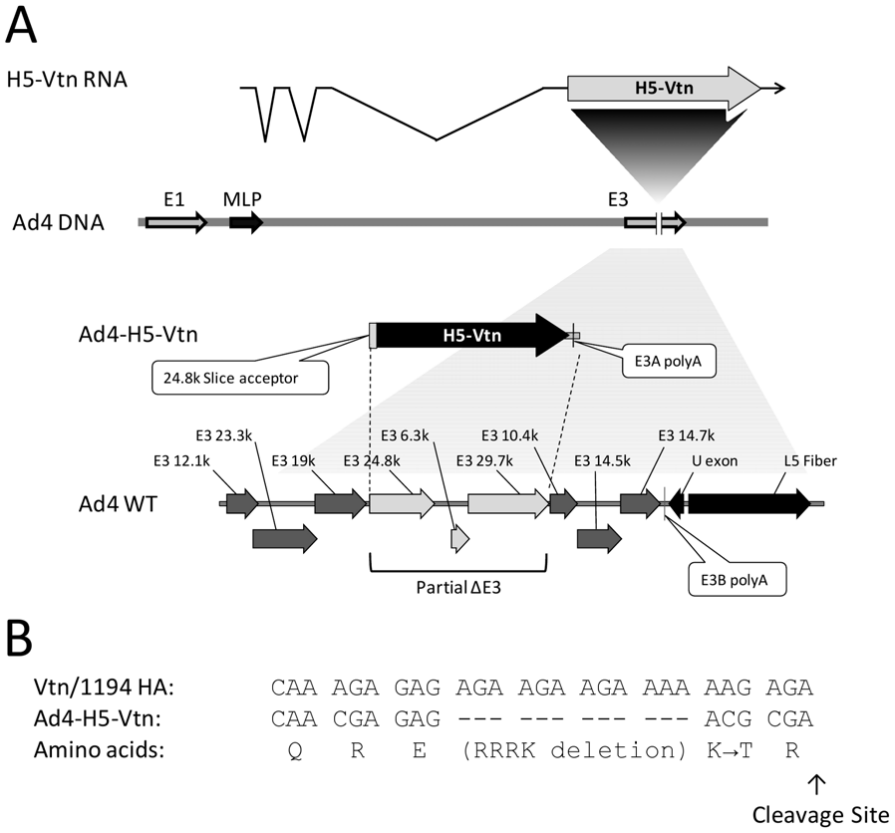
Ad4-H5-Vtn vector design. The H5HA native coding sequence, with the polybasic domain removed (B), was derived from A/Vietnam/1194/2004 influenza virus and inserted into the Ad4 virus E3 gene region. The Ad4 virus E3 24.8K, E3 6.3K and E3 29.7K genes were deleted to accommodate the HA transgene and the splice acceptor site of E3 24.8K was retained to drive expression of the HA transgene. The E3A polyadenylation signal sequence, derived from Ad5, was placed downstream of the HA coding sequence. The use of a shuttle plasmid encoding the H5HA sequence and the Ad4 plasmid to obtain the final vaccine product is described in Materials and Methods.

Bacterial recombination was used to generate the recombinant Ad4-H5-Vtn vaccine virus from a plasmid that contains the complete Ad4 genome derived from the Ad4 military vaccine (accession # AY594254). A shuttle plasmid was constructed with sufficient flanking Ad4 sequences to allow homologous recombination into the E3 region of the Ad4 plasmid. To incorporate the expression cassette for the H5-Vtn HA transgene (accession # EF541402), a partial deletion was made in the E3 which removed the coding regions for the unknown E3 24.8K, 6.8K and 29.7K ORFs (Figure 1A). The endogenous splice acceptor of E3 24.8K was left in place to facilitate the expression of H5-Vtn HA from the endogenous Major Late promoter (Figure 1A). The expression cassette consisted of several elements. The synthetic H5-Vtn HA gene was produced using the native DNA coding sequence but with the polybasic cleavage site removed (Figure 1B), consistent with the current vaccine strains, and included the 24.8K splice acceptor upstream of the coding region. The expression cassette included an Ad5-derived E3A polyadenylation signal sequence downstream of the HA coding sequence to define the boundary of early and late gene products from E3.

For homologous recombination; the full length Ad4 plasmid was linearized in the E3 region and the Ad4-flanked expression cassette fragment from the shuttle plasmid were combined and used to transform BJ5183 recombination-competent bacteria cells (Stratagene, La Jolla, CA). Clones were screened by restriction enzyme digestion and retransformed into Top10 cells for DNA production. The final clones of pAd4-H5-Vtn were rescreened by restriction digest and confirmed by DNA sequencing.

To produce virus for characterization; pAd4-H5-Vtn DNA was linearized with PacI to excise the bacterial sequences and transfected into A549 cells using Fugene HD transfection reagent under the standard recommended conditions. Once a cytopathic effect (CPE) was observed, generally 7–10 days after transfection, virus was harvested by scraping followed by 3 cycles of freeze-thaw cell disruption of the cell pellet. The lysate was clarified by centrifugation at 1,800× g for 10 minutes at 4°C and the supernatant collected. The virus was further expanded in A549 cells twice to generate sufficient virus for viral particle titer determination by HPLC [41]. Identity was confirmed by Western blot to evaluate transgene-specific protein expression and DNA sequencing of the E3 region. An additional expansion of the viral vector was accomplished using 10-Chamber cellSTACKS® (Corning®, Lowell, MA). The expanded virus was purified from the resulting crude lysate, following clarification, by anion exchange chromatography [41]. Typical virus yields were 1×10^13^ total purified Ad4-H5-Vtn viral particles from a single cellSTACK; with an average viral particle to infectious titer (TCID_50_) of 300∶1 or less. Purified virus was recovered in a Tris-glycerol buffer and stored at −80°C. Purified virus was used for all in vivo mouse experiments.

### Characterization of H5-Vtn HA expression

Translation of the H5-Vtn HA transgene was measured by Western Blot and flow cytometry. Ad4-H5-Vtn recombinant virus was serially-passaged 15 times in A549 cells to evaluate stability of transgene expression over time. Western blots were performed using Ad4-H5-Vtn infected A549 cells harvested when maximal CPE was observed. Cell lysate was produced using RIPA buffer (Thermo Scientific, Pittsburgh, PA) and assayed using Influenza A/Vietnam/1194/04 HA antiserum (NIBRG-14, NIBSC, U.K.) at a dilution of 1∶1,000. β-actin was assayed similarly using the Abcam antibody (Cambridge, MA) at a dilution of 1∶8,000.

For flow cytometry, A549 cells infected with Ad4-H5-Vtn were recovered after 48 hours and washed twice prior to labeling using an anti-H5HA specific monoclonal antibody (Advanced Immunochemical, Long Beach, CA). The cells were washed 2 times with FACS buffer, re-suspended in 100 µL goat anti-IgG PE secondary antibody (Southern Biotech, Birmingham, AL) and incubated in the dark at 4°C for 20 minutes. The cells were washed and re-suspended in 150 µL fixation buffer (CytoFix™, BD Biosciences) prior to analysis using a flow cytometer. Acquired sample data were analyzed using FlowJo software (Tree-Star, Inc, Ashland, OR).

### One-Step Growth Curve Analysis

Attenuation of Ad4 growth resulting from the insertion of the transgene was determined using single-step growth curves. There are three distinct sections to this procedure: 1) culture of human cell lines; 2) viral infection of human cell lines and 3) determination of viral growth by infectivity titering assay. Human cell lines (A549, MRC-5, HuTu80, HepG2 and H1299) were grown in various media as recommended by ATCC. Cells were harvested and plated (4×10^5^ cells/well in a 6-well plate, 2 mL volume/well) in culture media. Cells were infected when 50 to 70% confluence was achieved with Ad4-H5-Vtn or wild-type (Ad4wt) viruses at a concentration of 5×10^8^ vp/mL. The plates were incubated at 37°C and 5% CO_2_ for up to 72 hours with cells harvested at multiple time-points and virus released using 3 freeze-thaw cycles.

To measure viral replication, the virus supernatant from each time point was 10-fold serially diluted and 50 µL of each dilution used to infect a suspension of log phase A549 cells in growth media with 10% FCS before the mixture was plated into individual wells of 24-well plates (2×10^5^ cells/well; final virus dilutions, 1×10^−1^ to 1×10^−6^ in log 10 increments). The plates were incubated for 48 hours at 37°C and 5% CO_2_. Following incubation, the media was aspirated and cells were dried in the hood for 5 minutes. Cells were fixed with 0.5 mL ice-cold 100% methanol (Fisher Scientific, Waltham, MA). The plates were incubated for 15 minutes at −20°C followed by removal of methanol by aspiration. The plates were then washed 3 times with 0.5 mL PBS/1% BSA (Sigma-Aldrich, St. Louis, MO) followed by addition of 0.25 mL of hexon-specific primary mouse antibody (AbD Serotec, Raleigh, NC) diluted 1∶200 in PBS/1% BSA to each well and plates incubated for 1 hour at 37°C. Wells were then washed three times with PBS/1% BSA followed by addition of secondary rat anti-mouse HRP antibody (Invitrogen, Carlsbad, CA) diluted 1∶1000 in PBS/1% BSA and incubated for 1 hour at 37°C. Following incubation, plates were washed 3 times with PBS/1% BSA followed by addition of 0.25 mL DAB substrate (Invitrogen) to each well and plates incubated at RT for 10–20 minutes. Following incubation, the DAB substrate was aspirated and three fields containing 5–50 brownish/black spots (positive signal) were counted and averaged. The infectious units (IFU) per mL were calculated using the following formula: IFU/mL = (Spots/Field)×(Fields/Well)÷(Virus Volume (mL)×(Dilution Factor).

### Immunization of Mice and Influenza H5N1 reassortant Virus Challenge

To study the immunogenicity and efficacy of the Ad4-H5-Vtn vaccine, C57BL/6×BALB/c F1 mice (Charles River, Wilmington, MA) and the H5N1 reassortant virus (VNH5N1-PR8/CDC-RG, CDC Atlanta, GA) were used. Virus inoculums were administered intranasally (i.n.) to mice under light isoflurane (Baxter, Deerfield, IL) anesthesia.

To establish Ad4 pre-existing immunity, 50 µL containing 1×10^9^ vp Ad4wt virus was administered i.n. to mice. Ad4 immunity was confirmed by measuring Ad4 neutralizing antibody titers approximately one month following administration of Ad4wt virus. The Ad4-H5-Vtn recombinant vector vaccine virus was subsequently administered i.n. to groups of Ad4wt pre-immune and naïve mice (15 mice per group) at the following dosages; 1×10^9^, 1×10^8^, 1×10^7^ and 1×10^6^ vp per mouse.

As a positive control, animals were immunized subcutaneously with 15 µg of H5HA (A/Vietnam/1203/2004) protein (Protein Sciences, Meriden, CT) in PBS.

Each group was tested as follows: (1) Two mice (selected from random groups) were euthanized one month post Ad4wt immunization and pooled splenocytes used to complete Ad4 vector-specific IFN-γ ELISPOT assays; (2) 2 mice/group were euthanized one month post Ad4-H5-Vtn immunization and splenocytes used to complete H5HA-specific IFN-γ ELISPOT assays; (3) 2 mice/group were euthanized 5 days post H5N1 reassortant virus challenge and splenocytes used to complete IFN-γ ELISPOT assays. Additionally, at this same time, lungs were isolated and used to determine influenza viral titers; and (4) the remaining 10 mice were evaluated for weight change and survival for the duration of 14 days post challenge. Three random mice from each group were bled six weeks following Ad4-H5-Vtn vaccine immunization and again 5 days post influenza virus challenge to determine hemagglutination inhibition (HAI) antibody titers.

Mice were challenged i.n. with a lethal dose (5 MLD_50_ of virus in 50 µL) of the H5N1 reassortant virus 6 weeks post immunization with Ad4-H5-Vtn vaccine virus. This dose insured that a majority of the unvaccinated animals would not survive the viral challenge.

### Measurement of Ad4 Neutralizing Antibody Titer

Serial 5-fold dilutions of mouse immune serum (50 µL) were mixed with 3.3×10^6^ vp of Ad4wt virus (50 µL) and incubated at 37°C for 2 hour. Titering of the virus was performed as described for the one step growth curve analysis method. A 50% reduction of number of infected cells compared to un-neutralized virus alone equals the neutralization titer, expressed as reciprocal of dilution.

### Measurement of Ad4 and H5 Hemagglutinin-Specific T Cell Responses with an IFN-γ ELISPOT Assay

T cell responses specific for; (1) Ad4 virus and (2) a ‘pool’ of A/Vietnam/1194/2004 HA peptides were evaluated using an IFN-γ ELISPOT assay. Ad4 wt virus was heat inactivated for 1 hour at 72°C prior to use in the assay. The HA peptides in the pool consisted of: HA.156, seq. KSSFFRNVVWLIKKN; HA.241 seq. RMEFFWTILKPNDAI; HA.325 seq. NRLVLATGLRNSPQR; and HA.529 seq. IYQILSIYSTVASSLALAI. The four HA-derived peptides used in this study were selected based on predicted high binding affinity to HLA-DR molecules using algorithms from immunoepitope database (IEDB, www.iedb.org) [42]. All peptides were purchased from A & A Labs LLC (San Diego, CA). The purity of the peptides was substantiated by mass spectrometry and generally found to be greater than 95%. Peptides were dissolved in dimethyl sulfoxide at a concentration of 20 mg/mL, stored frozen at −20°C, and diluted with PBS before use.

Ninety-six well assay plates (MSIPS-4510, Millipore, Bedford, MA) were coated with 10 µg/mL of a monoclonal antibody specific for murine IFN-γ (clone AN18, Mabtech, Stockholm) by incubation overnight at 4°C. The antibody coated plates were washed 6 times with PBS and blocked by adding RPMI-1640 containing 10% bovine serum and incubated for 1 hour at 37°C. Unfractionated mouse splenocytes (5×10^5^) were added to triplicate wells containing antigen (2×10^9^ Ad4wt vp per well or 0.4 µg of each HA-derived peptide per well) in a total volume of 0.2 mL per well. Plates were incubated for 24 hours at 37°C. Wells were then washed 6 times with PBS containing 0.05% Tween 20 (Sigma) followed by a 1 hour incubation with 100 µL of biotin-conjugated monoclonal antibody (1 µg/mL final) specific for murine IFN-γ (clone R4-6A2, Mabtech) diluted in PBS containing 0.5% BSA (Sigma). Plates were washed 6 times with PBS 0.05% Tween 20 before adding 100 µL of an avidin-peroxidase complex (eBioscience, San Diego, CA), diluted in PBS containing 0.5% BSA, was added to each well and incubated for 1 hour at room temperature. The plates were washed 6 times with PBS containing 0.05% Tween 20 and 3 times with PBS. AEC peroxidase substrate solution (100 µL) (Vector Laboratories Inc., Burlingame, CA) was used as the color development substrate, which was stopped after 5 minutes with the addition of water. Spots were counted using a CTL S5 Core plate reader (Cellular Technologies Ltd, Shaker Hts, OH) and data defined as numbers of spot forming cells (SFC) per 1×10^6^ splenocytes ± the standard deviation for each peptide. The limit of quantitation for the ELISPOT assay was established as 50 SFC/10^6^ splenocytes, a cut-off similar to values described by other investigators [43].

### Hemagglutination Inhibition (HAI)

HAI was performed by incubating with 4 HA units of the H5N1 reassortant virus in the presence of serially diluted sera, previously treated overnight with receptor destroying enzyme, RDE (Accurate Chemical and Scientific, Westbury, NY) to destroy heat stable non-specific inhibitors in duplicate wells of a 96 well plate for 30 minutes at RT. Chicken RBCs (Charles River Laboratories, Wilmington, MA) were then added and plates incubated for an additional 30 minutes. Plates were tilted and then visually inspected for agglutination. The reciprocal of the highest dilution of antibody that causes inhibition of hemagglutination was considered the HAI titer.

### Influenza Viral Titer Measurement from Mouse Lungs

To assess the clearance of H5N1 reassortant influenza challenge virus from the lungs, 2 mice from each group were euthanized 5 days post viral challenge and lungs were harvested for use. Lungs were snap frozen on dry ice with ethanol and stored at −70°C. Lungs were thawed and then homogenized with a FastPrep-24 tissue homogenizer (VDI 12 Tissue Homogenizer, VWR International) in 1 mL PBS per lung. Tissue homogenates were clarified by centrifugation (10,000× g), the supernatant was filtered and 6 replicates of 5-fold serial dilutions of supernatant were added to 96-well plates containing MDCK cells (ATCC #CCL-34). Plates were cultured at 37°C and 5% CO_2_. Following 18 hour incubation, the presence of anti-influenza nucleoprotein NP was determined using a modified ELISA protocol. Media was discarded and cells were washed once with PBS then fixed with 80% methanol. Wells were then washed with PBS containing 0.05% Tween 20 and anti-NP antibody (Chemicon, Temecula, CA) added followed by 1 hour incubation at room temperature. After thorough washing, NP-specific antibodies were detected by incubating for 1 hour with a goat anti-mouse IgG HRP-coupled secondary antibody (Invitrogen). The plates were developed with TMB HRP substrate reagent, stopped with acid, and absorbance measured on a PolarStar plate reader at 450 nm. Lung viral titers were calculated as TCID_50_/mL according to the Reed and Muench method [44].

### Statistical Analysis

ELISPOT: the Student *t* test was used to compare the immune response between immunized vs. naïve (non-immunized) mice as well as for the impact of pre-existing immunity. A *p* value of <0.05 was used as the indication of significant responses. Additionally, to be considered a positive response, a minimum of 50 IFN-γ SFC had to be achieved.

Survival: Statistically significant differences in survival times were established using the Fisher's exact test analysis. Differences were considered statistically significant if the *p* value was <0.05.

## Results

### Ad4-H5-Vtn Virus Generation

The Ad4-H5-Vtn virus was generated in A549 cells, with CPE appearing only a few days delayed compared to an Ad4wt control. Extraction of the viral DNA and restriction digestion mapping with several combinations of enzymes confirmed at a gross level that the viral genome was intact without major deletions or insertions (data not shown). PCR across the E3 region (including the expression cassette) generated a DNA product of the correct size and sequencing of the PCR product confirmed the sequence of the recombinant E3 region (data not shown). Similar analysis was performed after 5, 10 and 15 serial passages in A549 cells and confirmed that the viral genome was genetically stable (data not shown).

**Figure 2:**
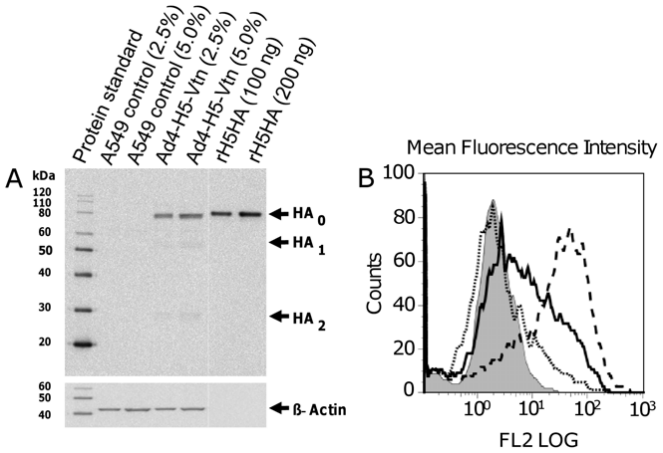
Ad4-H5-Vtn virus infection of A549 cells induced H5 HA protein expression as detected by Western blot and flow cytometry analysis. In the case of western blot analysis (A), A549 cells were infected in suspension with clarified crude lysate of Ad4-H5-Vtn virus from the 14^th^ passage. The cell extracts, 2.5% and 5%, were subjected to SDS-PAGE in 4–12% Bis-Tris gel, blotted onto nitrocellulose membrane and probed with anti-1194 H5HA polyclonal sheep antibody. Lysates as indicated, from uninfected A549 cells were used as negative control. Recombinant His tagged H5HA1203/2004 100 ng and 200 ng proteins were used as positive controls. β-actin was used as loading control. HA_0_ refers to full length H5HA; HA_1_ and HA_2_ represent the proteolytically cleaved form of HA. In the case of flow cytometry (B), A549 cells were infected with a dose titration (vp/mL) of the Ad4-H5-Vtn vector: dotted line, 5×10^6^; solid line, 5×10^7^; dashed line, 5×10^8^. Infected cells were removed and subsequently incubated with primary anti-H5HA and secondary goat anti-IgG PE antibodies. A negative control, A549 cells infected with Ad4wt virus (5×10^8^ vp/mL) was included; grey fill.

Protein expression of H5-Vtn HA was evaluated from A549 cells infected with the recombinant virus by both western and flow cytometry. Western analysis confirmed that Ad4-H5-Vtn infected A549 cells robustly expressed HA protein of the correct 80 kDa size similar to purified recombinant (r)H5 protein positive controls (Figure 2A). Minor bands were also observed corresponding to HA_1_ and HA_2_, cleavage products of full-length HA_0_. Specificity of the antibody was affirmed by the lack of cross reactivity in the uninfected A549 cells negative control.

Flow cytometry confirmed that the expressed HA was present on the cell surface of Ad4-H5-Vtn-infected A549 cells (Figure 2B) and that mean fluorescence intensity (MFI) increased in a dose-dependent manner. Specifically, the following MFIs were observed: 5×10^6^ vp/mL (6.0 MFI); 5×10^7^ vp/mL (13.1 MFI); 5×10^8^ vp/mL (34.0 MFI). As a negative control, A549 cells infected with Ad4wt virus resulted in an MFI of 2.2.

### Ad4-H5-Vtn Virus Growth is Attenuated Relative to Ad4wt Virus

**Figure 3:**
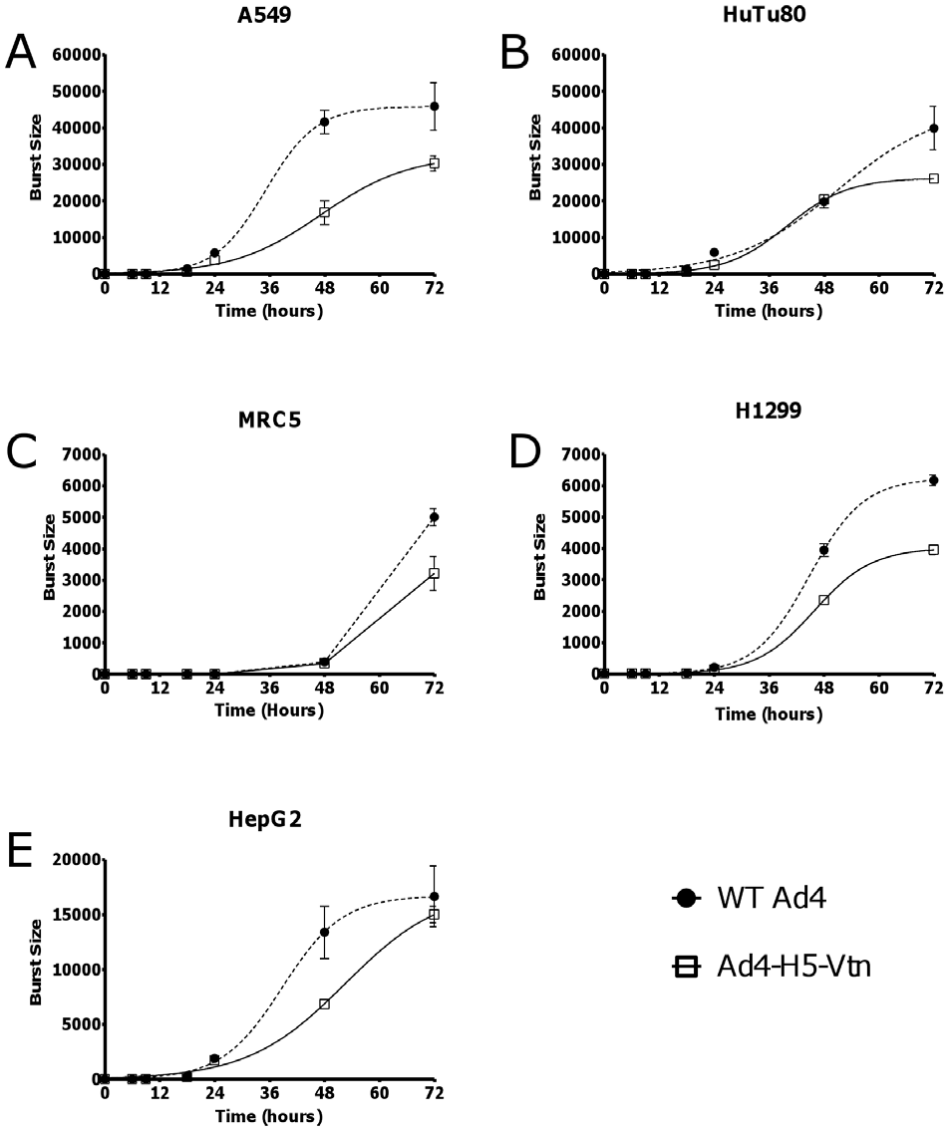
Ad4-H5-Vtn virus growth is attenuated in various human cell lines versus Ad4wt virus. Growth of Ad4-H5-Vtn virus was compared to growth of Ad4wt virus in several human cell lines: A549 a lung carcinoma cell line (A); Hu Tu 80 a duodenum adenocarcinoma cell line (B); MRC-5 an embryonic lung fibroblast cell line (C); H1299 a lung carcinoma line (D); and HepG2 a hepatocellular carcinoma cell line (E). Virus infection and cell growth conditions are described in Materials and Methods.

Conventional animal vaccine safety studies are not informative for the Ad4-H5-Vtn vaccine as Ad4 is species specific and will not replicate in non-human species. Thus, we compared the growth of Ad4-H5-Vtn virus to Ad4wt virus in several human cell lines; A549, Hu Tu 80, MRC-5, H1299 and HepG2. Growth and replication rate of Ad4-H5-Vtn virus were similar or attenuated relative to Ad4wt virus. Specifically, when evaluating the 48 hour time points following cell infection, the burst sizes were comparable (Hu Tu 80, MRC-5) to 1.8- to 2.7-fold lower (H1299, HepG2, A549) for Ad4-H5-Vtn virus in the cell lines tested (Figure 3). At the 72 hour time point, Ad4-H5-Vtn virus was approximately 1.1- to 1.7-fold lower in burst size relative to Ad4wt for all cell lines tested. In no case was growth of the Ad4-H5-Vtn virus higher than that of Ad4wt virus.

### Vaccine-Induced H5HA-specific Humoral Response in the Presence and Absence of Pre-existing Ad4-specific Immunity

**Figure 4:**
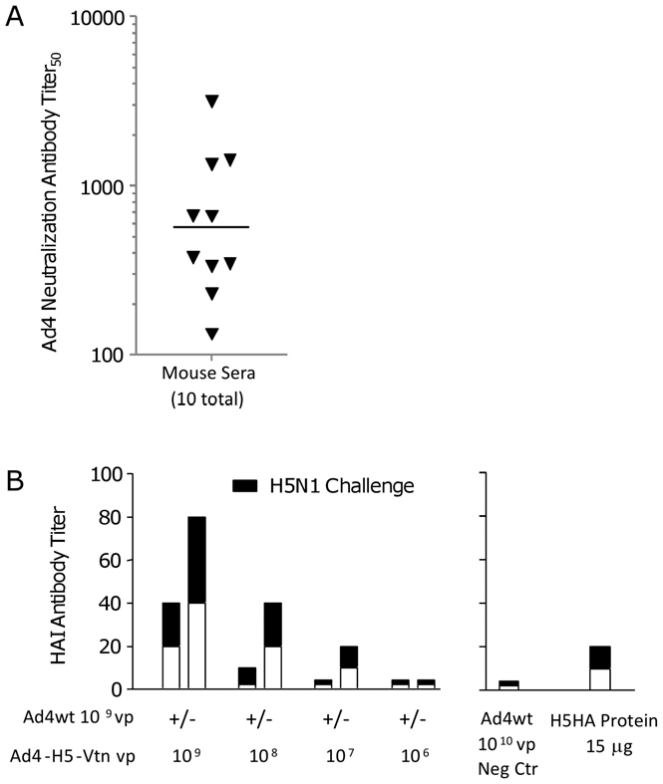
Vaccine-induced H5HA-specific humoral response in the presence and absence of pre-existing Ad4-specific immunity. Mice were immunized i.n. with 1×10^9^ vp of Ad4wt virus per mouse to establish pre-existing immunity to the vector. Four weeks following the immunization, ten individual mice were bled and Ad4-specific neutralizing antibody titers were determined (A). Mice immunized with Ad4wt virus and naïve mice were subsequently immunized i.n. with a dose titration of the Ad4-H5-Vtn vaccine; 1×10^9^, 1×10^8^, 1×10^7^ and 1×10^6^ vp per mouse and bled 6 weeks after vaccine immunization and again 5 days later following H5N1 reassortant virus challenge to determine HAI antibody titers (B). The immune responses are represented by post-Ad4-H5-Vtn vaccine immunization (open bar) and post-H5N1 reassortant challenge (cumulative of open and black fill bar). Three mice from the group were bled and sera pooled to determine HAI antibody titers.

To evaluate the effect of pre-existing immunity to Ad4wt virus on vaccine potency, mice were immunized i.n. with 1×10^9^ vp of Ad4wt virus and one month later Ad4-specific neutralizing antibody titers and cellular immunity were determined. Immune sera from 10 immunized mice were collected and Ad4-specific neutralizing antibody titers measured. Titers ranged from 133 to 3,140 with a geometric mean of 569 (Figure 4A). Sera from naïve mice exhibited values of <10 for neutralizing antibody titers (data not shown). Mice demonstrating pre-existing Ad4wt immunity and naïve mice were subsequently used for Ad4-H5-Vtn vaccine immunizations. Naïve mice exhibited higher HAI antibody titer trends relative to mice with pre-existing immunity to Ad4wt vector: 40 vs. 20; 20 vs. <10; and 10 vs. <10 for 10^9^, 10^8^ and 10^7^ vp Ad4-H5-Vtn vaccine doses, respectively (Figure 4B, open bars). Following the H5N1 reassortant virus challenge, HAI responses were typically boosted higher by one dilution (Figure 4B, black fill bar). The lowest dose of vaccine, 10^6^ vp, was not immunogenic, i.e., <10 HAI antibody titer. As comparator controls, mice immunized with 15 µg of recombinant H5HA protein induced 10 and 20 HAI antibody titers pre- and post-H5N1 reassortant virus challenge. Significant HAI antibody titers, 5 days post virus challenge, were not detected in mice immunized with only Ad4wt virus and subsequently challenged with H5N1 reassortant virus.

### Vaccine-Induced H5HA-specific Cellular Response in the Presence and Absence of Pre-existing Ad4-specific Immunity

**Figure 5:**
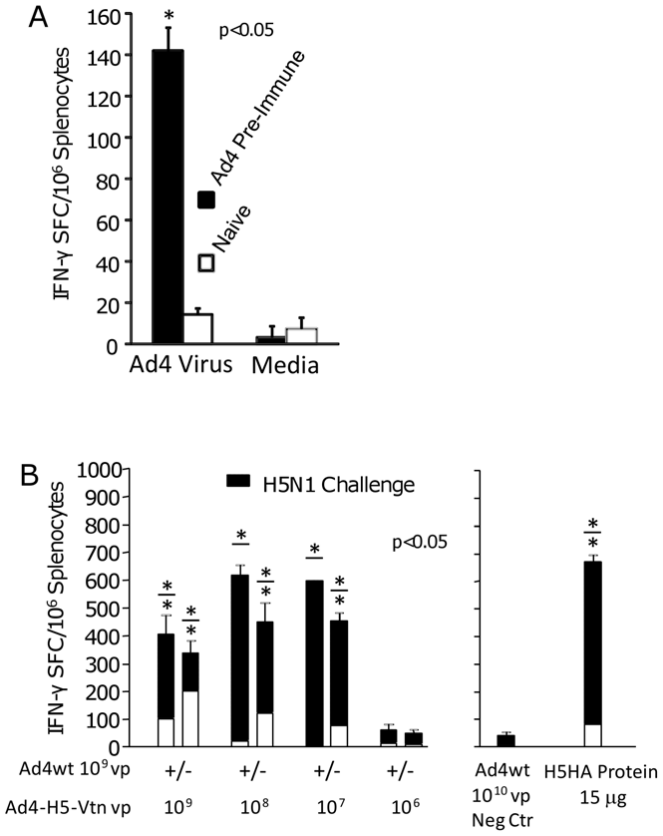
Vaccine-induced H5HA-specific cellular response in the presence and absence of pre-existing Ad4-specific immunity. Mice were immunized i.n. with 1×10^9^ vp of Ad4wt virus per mouse to establish pre-existing immunity to the vector as previously stated. Two mice were sacrificed and splenocytes pooled to determine Ad4wt virus-specific cellular immunity, as assayed by IFN-γ ELISPOT (A). Two mice were also sacrificed 6 weeks after vaccine immunization and again 5 days later following H5N1 reassortant virus challenge and splenocytes pooled to determine H5 HA-specific cellular immunity evaluated by IFN-γ ELISPOT specific for four H5HA-derived 15-mer peptides, stimulating peptides for ELISPOT response (B). In the case of part (B), the immune responses are represented by post-Ad4-H5-Vtn vaccine immunization (open bar) and post-H5N1 reassortant challenge (cumulative of open and black fill bar). Mice pre-treated with Ad4wt virus and challenged with H5N1 reassortant virus demonstrated no detectable H5HA-specific cellular responses 5 days post-influenza virus challenge. An asterisk * denotes significant IFN-γ responses, *p*<0.05. In the case of two asterisks (*/*) associated with a bar, the bottom and top asterisks refer to post-immunization and post-reassortant H5N1 virus challenge, respectively.

Significant Ad4-specific cellular immunity (140 IFN-γ spot forming cells (SFC) per 1×10^6^ splenocytes) against heat-inactivated Ad4wt virus was observed compared to <50 IFN-γ SFC using splenocytes from naive mice (Figure 5A). Pre-existing Ad4wt immunity also affected vaccine induction of cellular immunity, specific for a pool of four 15-mer peptides derived from the A/Vietnam/1194/2004 HA protein (Figure 5B, open bar). Generally, naïve mice exhibited significantly higher H5HA-specific cellular immune responses (SFC) relative to mice with pre-existing immunity to Ad4wt vector: 200 vs. 100; 120 vs. <50; and 80 vs. <50 for 10^9^, 10^8^ and 10^7^ vp Ad4-H5-Vtn vaccine doses, respectively (Figure 5B, open bar). Cellular responses were augmented 5 days following H5N1 reassortant virus challenge. In the presence or absence of pre-existing Ad4wt immunity, 10^7^, 10^8^, 10^9^ vp doses of Ad4-H5-Vtn vaccine induced between 300 and 600 IFN-γ SFC (Figure 5B, black fill bar). It should be noted that in the case of pre-existing Ad4wt immunity and vaccine doses of 10^7^ and 10^8^ vp, minimal non-significant IFN-γ responses of <50 SFC were boosted to >500 SFC following H5N1 reassortment virus challenge suggesting that even the lower vaccine doses primed for cellular responses. The 10^6^ vp dose was not immunogenic, i.e., <50 SFC. As comparator controls, mice immunized with 15 µg of recombinant H5HA protein induced 80 and 680 IFN-γ SFC post-immunization and post-H5N1 reassortant virus challenge, respectively. Significant HA-specific cellular responses, 5 days post H5N1 reassortant virus challenge, were not detected in mice immunized with only Ad4wt virus.

### Efficacy of the Ad4-H5-Vtn Vaccine: Weight Loss, Survival and Reduction of Lung Influenza Virus Titers

**Figure 6:**
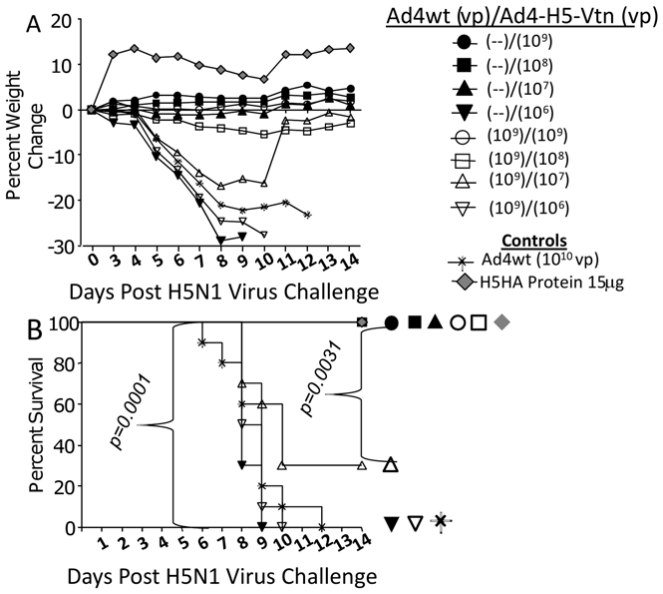
Mice immunized with Ad4-H5-Vtn vaccine lost no weight, survived a lethal H5N1 reassortant viral challenge and presented with a reduction of H5N1 reassortant virus in the lungs. Groups of mice were immunized with Ad4wt virus to establish pre-existing immunity as described previously in Materials and Methods section. Mice were subsequently immunized intranasally with a dose titration of the Ad4-H5-Vtn vaccine. Six weeks following Ad4-H5-Vtn vaccine immunization, the mice were challenged with a lethal dose of H5N1 reassortant virus. If animals were recorded to have lost 20% or more of their original weight for two days in a row they were euthanized. Weights of the mice were evaluated daily (A), and survival of mice were evaluated over a 14 day period (B).

Weight loss, survival and reduction of lung influenza virus titers were vaccine dose dependent following lethal H5N1 reassortant virus challenge. Weights of the animals were measured for 14 days following H5N1 reassortant challenge (Figure 6A). In animals without pre-existing Ad4-specific immunity little to no weight loss was observed at the 10^7^, 10^8^ and 10^9^ vp Ad4-H5-Vtn vaccine doses. Ad4wt pre-existing immunity could be overcome and prevent weight loss when using the higher Ad4-H5-Vtn vaccine dose of 10^9^ vp. Mice immunized with recombinant H5HA protein lost no weight while Ad4wt immunized mice succumbed to disease.

Survival of mice is shown in Figure 6B. Groups of mice receiving 10^8^ and 10^9^ vp of vaccine were completely protected (*p* = 0.0001). Note that pre-existing immunity to Ad4wt virus did not affect animal survival at these doses. However, at lower vaccine dose of 10^7^ vp, the vaccine was not as effective in the presence of pre-existing immunity (*p* = 0.0031). The lowest vaccine dose of 10^6^ vp was not protective regardless of Ad4 pre-immune status and the use of Ad4wt virus without the H5HA transgene did not protect animals.

**Figure 7:**
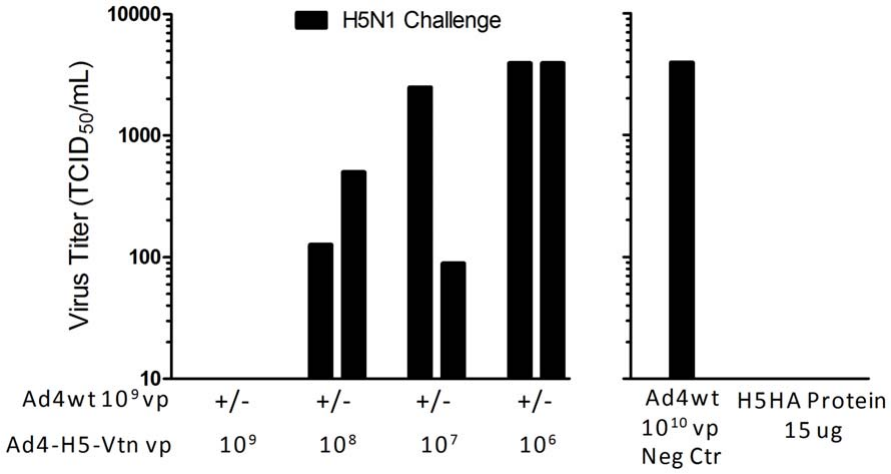
Mice immunized with Ad4-H5-Vtn vaccine presented with a reduction of H5N1 reassortant virus in the lungs. Groups of mice were immunized with Ad4wt virus to establish pre-existing immunity as previously described in Materials and Methods section. Mice were subsequently immunized intranasally with a dose titration of the Ad4-H5-Vtn vaccine. Six weeks following Ad4-H5-Vtn vaccine immunization, the mice were challenged with a lethal dose of H5N1 reassortant virus. Lungs were recovered from a subset of mice 5 days post-challenge to determine influenza-specific viral titers.

Lungs from two mice representing each group were obtained 5 days post-H5N1 reassortant virus challenge to evaluate influenza virus titer (Figure 7). Protection against influenza virus challenge was most evident in the mice immunized at the higher vaccine dose of 10^9^ vp and mice immunized with H5HA protein where a measurable influenza virus titer was not obtained from lungs. In contrast, lungs from negative control mice immunized with Ad4wt virus and mice immunized with the lower vaccine dose of 10^6^ vp generated a virus titer of approximately 4,000 TCID_50_/mL. At the vaccine dose of 10^8^ vp, an approximate 8- to 31-fold reduction in virus titer was observed. Effects of pre-existing immunity to Ad4wt virus was most evident at the lower vaccine dose of 10^7^ vp where an approximate 44-fold and 1.5-fold reduction of virus was observed in the absence and presence of pre-existing immunity.

## Discussion

The efforts of the study were to design, produce, and evaluate for safety, immunogenicity and efficacy a recombinant replicating Ad4 virus encoding the H5HA gene from avian influenza H5N1 strain, A/Vietnam/1194/2004, as a prototype vaccine. The Ad4-H5-Vtn virus readily infected A549 cells and supported robust expression of H5 HA protein, as demonstrated using western blot and flow cytometry analyses. The Ad4-H5-Vtn virus was attenuated in its growth capacity supporting an acceptable safety profile.

Although human Ad4 viruses will not replicate, i.e., generate a productive infection, in cells of nonhuman origin [15], [18], [45], [46], [47], they will infect nonhuman cells presumably through the evolutionarily conserved cocksackie and adenovirus receptor (CAR) [48]. The infected cells of non-human origin will support transcription and translation of both Ad4 vector and transgene sequences. We took advantage of this property to assess vaccine immunogenicity and efficacy using a mouse model as a prelude to Phase 1 clinical trial vaccine evaluation. The vector vaccine virus induced H5HA-specific HAI antibody and cellular immune responses after a single i.n. immunization of mice even in the presence of pre-existing Ad4-specific neutralizing antibody. The immune responses of vaccinated mice challenged with the H5N1/PR8 reassortant virus five days post influenza virus challenge were characterized by rapid increases of both H5HA-specific antibody and cellular immune responses. We did not assess the individual contribution of CD8^+^ and CD4^+^ T cells to the overall cellular response. A study by Hoelscher and colleagues using a similar vaccine approach demonstrated that mice immunized with a recombinant adenovirus H5 HA vaccine induced an HA-518-epitope-specific IFN-γ-secreting CD8^+^ T cell response [30]. In this study, T cell responses specific for HA-derived 15-mer peptides were measured. The peptides were selected on the basis of predicted binding to HLA-DR molecules and therefore are predicted to measure predominately CD4^+^ T cell responses. However, to formally address this issue, HA-specific cellular responses induced by the Ad4-H5-Vtn vaccine will be evaluated specifically for contributions of CD4^+^ and CD8^+^ T cell subsets in the current ongoing Phase 1 trial. Vaccine-induced immune responses afforded a significant level of protection against lethal H5N1 reassortant virus challenge as measured by reduced weight loss, survival and reduced influenza virus replication in the lungs. It should be noted that a single time point of 5 days post influenza virus challenge was evaluated for virus titer in the lungs. It was concluded that mice immunized with the higher doses of Ad4-H5-Vtn vaccine had a significantly lower virus titer relative to mice immunized with the Ad4wt control. It is not known whether the reduction in virus titer was due to accelerated viral clearance or alternatively an initial lower virus load in the immunized animals. Of particular note is that efficacy was also observed despite the absence of measurable immune responses, which were characterized with anamnestic responses indicating the vaccine had primed the immune system. The Ad4-H5-Vtn vaccine induction of H5HA-specific humoral and cellular immunity generally correlated with efficacy. Pre-existing immunity to the Ad4wt vector did have an effect on both immunogenicity and efficacy but the effect was more pronounced at the lower vaccine doses suggesting that pre-existing immunity to the Ad vector may be overcome by using higher vaccine doses.

We selected the replicating Ad4 virus and mucosal route of administration for several reasons including: 1) safety and efficacy as documented by the U.S. Military Ad4 vaccine program; 2) potential use of lower doses due to replication competence; 3) induction of potent innate and adaptive immune system responses; 4) induction of immunological memory 5) a means to minimize effects of pre-existing immunity to the vector; and 6) capacity via molecular methods to readily generate new Ad4 vector-based vaccines expressing disease transgenes of interest [8], [21], [28], [37], [38], [39], [40], [49], [50], [51], [52], [53], [54], [55], [56], [57], [58]. In addition, we have the capacity to quickly change the HA to address an emerging H5N1 threat from around the globe. Several agencies including the World Health Organization monitor cases around the world and are able to detect new species in both domestic and wild bird populations as well as any documented human cases.

We designed the influenza vaccine using an insertion site for HA in the non-essential E3 region to preserve virus replication in human cells. In contrast, a majority of recombinant Ad vector vaccine development to date has relied on Ad5 vector which will not replicate in humans due to modification of the replication essential E1 region.

There are possible limitations for the use of Ad-based vaccines, most notably potential lack of vaccine potency due to pre-existing immunity. Pre-existing immunity may be derived from natural infection or repeated Ad-based vaccine immunization. As reported, the overall prevalence for Ad4 neutralizing antibodies is approximately 30% [59]. We evaluated the effects of pre-existing immunity experimentally. Our initial task was to establish Ad4-specific pre-existing immunity in mice to a level typically observed in man. Lyons and colleagues reported in a recent study of the Ad4 (and Ad7) vaccine during a period ongoing Ad4wt transmission and disease, that 36 individuals with pre-existing immunity to Ad4 virus demonstrated neutralizing antibody titers corresponding to a range of 12–724, with a geometric mean of 78 [53]. In the mouse studies, described herein, the Ad4-specific neutralizing antibodies induced following Ad4wt immunization were higher with a range of 133–3140, and a geometric mean of 569. Thus, it is likely that Ad4 responses induced in our animal system were at least comparable or higher than those observed in man. Using this experimental variable we observed the administration of higher vaccine doses and mucosal delivery are sufficient to overcome the effects of pre-existing immunity, an observation consistent with numerous reports [49], [51], [52], [54], [55], [57], [58]. Data from several human studies support this concept. For example, orally administered replicating rotavirus vaccines induce an antibody response in infants who have pre-existing rotavirus antibody [60]. Recent experience with the Ad serotype 4 (Ad4) and Ad serotype 7 (Ad7) vaccines suggests that pre-existing Ad4 or Ad7 neutralizing antibody did not prevent replication or an immune response induced by the vaccine, although it may have diminished it [53].

In summary, these data support further evaluation of the live, replication-competent, recombinant Ad4 candidate vaccine, which expresses the HA of H5N1 A/Vietnam/1194/2004 influenza virus in a Phase 1 clinical trial. It is hypothesized that an orally administered enteric-coated capsule containing Ad4-H5-Vtn virus will release the virus in the intestine, replicate, express H5HA, and elicit a humoral and cellular immune response that will be protective against H5N1 influenza. A first-in-man, Phase 1 double-blind, placebo controlled ascending dosage study is currently in-progress in which 166 volunteers (vaccine∶placebo = 3∶1) have been given escalating dosages of this Ad4-H5-Vtn vaccine (10^7^, 10^8^, 10^9^, 10^10^ and 10^11^ vp total dose). The final drug product was generated by lyophilizing purified Ad4-H5-Vtn (produced in MRC-5 under GMP conditions) into a dry powder form. The dry virus was then adjusted to the desired dosage concentration using dry sucrose excipient, filled into hydroxypropylmethylcellulose (HPMC) capsules, and enteric-coated. The primary objectives of the clinical study are to evaluate safety and humoral immunogenicity following oral administration of the vaccine. Additional objectives of this study are to evaluate the mucosal and cellular immune responses to the influenza H5HA antigen, investigate the replication and excretion (shedding) of the Ad4-H5-Vtn virus, and evaluate the effect of pre-existing immunity to Ad4 on these parameters.
